# Experiences of complementary and alternative medicine in patients with inflammatory bowel disease – a qualitative study

**DOI:** 10.1186/1472-6882-14-407

**Published:** 2014-10-22

**Authors:** Annelie Lindberg, Bjöörn Fossum, Per Karlen, Lena Oxelmark

**Affiliations:** Division of Gastroenterology, Department of Clinical Sciences, Karolinska Institutet, Danderyd Hospital, SE-18288 Stockholm, Sweden; Department of Nursing, Sophiahemmet University, Stockholm, Sweden; Department of Neurobiology, Care Sciences and Society, Division of Nursing, Karolinska Institutet, Stockholm, Sweden; Institute of Health and Care Sciences, The Sahlgrenska Academy, University of Gothenburg, Gothenburg, Sweden

**Keywords:** CAM, Healthcare professionals, IBD, Patients’ experience, Qualitative research

## Abstract

**Background:**

The use of Complementary and Alternative Medicine (CAM) in Inflammatory Bowel Disease (IBD) is increasing. Although CAM often improves patients’ well-being, it can also lead to side-effects and interactions with conventional medications. Research on patients with IBD in Sweden who have experiences of CAM is sparse. More studies are needed to enhance awareness of and improve communication about CAM. The aim of this study was to describe experiences of CAM in the healthcare context reported by patients with IBD.

**Methods:**

Fifteen patients with IBD, eight with Crohn’s disease (CD) and seven with ulcerative colitis (UC), were recruited. Semi-structured qualitative interviews were conducted and qualitative content analysis was performed.

**Results:**

The analysis revealed the theme Knowledge and communication lead to participation in the area of CAM based on three categories; CAM use, Communication and Self-care. Patients with IBD wanted to be asked about CAM to be able to start a dialogue, as some perceived being treated in a disparaging manner and not taken seriously when raising the subject. Healthcare professionals (HCPs) need to be aware of this in order to meet and understand patient needs. Patients with IBD found it easier to communicate about CAM with the IBD nurses than physicians and dietary changes was one important CAM treatment.

**Conclusions:**

The finding that it was easier to discuss CAM with nurses than physicians emphasizes the important role of the IBD nurse in communication and monitoring patients’ CAM use. Patients wanted to be asked about CAM to be able to start a dialogue, as some perceived not taken seriously when raising the subject. Furthermore, HCPs need to understand that many patients with IBD regard dietary changes as an important part of CAM treatment. Further research in these areas is needed.

## Background

The World Health Organization (WHO) defines Complementary and Alternative Medicine (CAM) as a broad set of healthcare practices, systems and products that are not generally considered part of the country’s own tradition and are not integrated into the main healthcare system [1]. What constitutes CAM differs from country to country. CAM can be categorized into five domains: whole medical systems, mind-body interventions, biologically-based therapies, manipulative and body-based methods and energy therapies. Whole medical systems comprise theories and practices such as homeopathic medicine, Traditional Chinese Medicine and Ayurveda. Mind-body interventions strengthen communication between the mind and body, for example meditation, prayer and healing. Biologically-based therapies involve substances found in nature, including dietary supplements and herbal products. Manipulative and body-based methods employ human touch to move or manipulate a specific part of the body, for example, chiropractic therapy and massage. Energy therapies use energy fields in the body to promote health and healing, for example qi gong, tai chi and magnet therapy [2]. Complementary medicine refers to the use of CAM in combination with conventional medicine, while alternative medicine implies the use of CAM instead of conventional medicine. Integrative medicine combines both conventional and evidence based CAM treatment [2].

CAM use has increased in the Scandinavian countries [3]. In Norway and Denmark, half and one third of hospitals, respectively, offer CAM to patients [4]. The population of Sweden is 9.7 million inhabitants, of whom 2.1 million live in the capital Stockholm. CAM use in Stockholm has increased from 22% in 1980 to 49% in 2002 [5]. Although CAM is becoming more common in all parts of Sweden, studies have mostly been published in less renowned journals. The Swedish Research Council for Health, Working Life and Welfare (FAS) has highlighted the importance of determining CAM use. In view of the increasing prevalence of CAM, more evidence based research is required [5]. In Sweden, healthcare is financed by taxes and all citizens have the right to healthcare on equal terms [6]. There is no national CAM policy in Sweden, and it is not officially approved in healthcare or in the education system, thus policy development is essential [7]. Interest in CAM is growing among patients with chronic diseases [8, 9] including inflammatory bowel diseases (IBD) [10–12].

IBD is a group of inflammatory conditions of the colon and small intestine, most commonly ulcerative colitis (UC) and Crohn’s disease (CD). They are chronic, intermittently relapsing diseases with inflammation and ulceration of the digestive tract. The symptoms range from mild to severe and include abdominal cramps with frequent diarrhoea, bowel urgency and occasionally fever [13]. IBD can be severely disabling and affect quality of life as well as the ability to live a normal life [14–16]. The medical treatment may also have a major impact on patients’ health-related quality of life (HRQOL) and the main challenge is to combine therapies with minimal side effects and optimal quality of life [17, 18]. The cause of IBD is unknown and there is no medical cure, although several therapeutic advances have been made in recent years. The complexity of IBD treatment has increased, as the use of new immunomodulation agents [17, 19, 20]. IBD can be associated with liver and biliary tract disorders [21, 22], which makes it especially important to be aware that some CAM treatments such as herbal medicinal products [23] and noni juice (Morinda citrifolia) may be associated with liver toxicity [24, 25]. Massage, natural remedies, relaxation, yoga and counselling has been shown to be the most commonly used CAM therapies in Swedish IBD patients [26]. Reasons for CAM use may be lack of efficacy of conventional IBD therapy [12, 27], steroid treatment side-effects [28], stress and wish for a holistic approach [29] or greater control over the disease [30].

Research has revealed communication gaps between physicians and patients with regard to CAM [31–33] and few clinicians routinely initiate discussions about it with patients [33, 34]. Communication style can have an impact on health outcomes and patients of physicians who employ a participatory decision-making style have better health outcomes [35, 36] and may also be more willing to mention their CAM use [37].

There is a growing need to educate HCPs about CAM, as knowledge of possible effects and interaction risks is essential [38]. HCPs have expressed the need for CAM education in order to provide optimal advice [39–41]. To understand the needs of patients with IBD it is essential for Healthcare professionals (HCPs) to have knowledge of their experiences and expectations of CAM. The aim was to describe experiences of CAM in the healthcare context reported by patients with IBD.

## Methods

This study was based on qualitative interviews. The qualitative method provides a deeper understanding of a phenomenon by focusing on words in the collection and analysis of data [42, 43].

### Participants

The respondents comprised 15 patients with IBD, 8 with CD and 7 with UC, recruited from 2 out-patient IBD clinics in one urban area of Sweden. The respondents were 9 women and 6 men, ranging in age from 20–80 years (mean 45 years), with a disease duration of 2–34 years (Table 1). All those invited agreed to participate in the study. Purposeful sampling was used to obtain as much variation as possible in terms of experience and to gain insight into the phenomenon of CAM as experienced by patients with IBD [43]. The purposeful sampling was based on the following variables: diagnosis, gender, age and disease duration.

**Table 1:** **Demographic data**

	Total
Respondents (n)	15
Female/male (n)	9/6
Age, mean years (range)	45 (20–80)
CD/UC	8/7
Disease duration, mean years (range)	14 (2–34)
Education – less than 12 years/more than 12 years	10/5

### Data collection

All the interviews were conducted in a quiet, private room at the out-patient clinic. Before the interview the respondents provided their written informed consent. The interviews lasted from 15–50 minutes.

### Ethical considerations

The study was approved by the Regional Ethical Review Board in Stockholm, Sweden (no. 2008/4:6). All respondents were assured of confidentiality, and informed that participation was voluntary and that they could withdraw at any time.

### Data analysis

A qualitative content analysis method was used in accordance with Krippendorf [42]. Content analysis is a step-wise technique for analysing the content of a text in its context [42, 43]. The transcripts were analysed using manifest and latent content analysis. The former refers to the obvious meaning of the text while the latter comprises interpretive reading in order to capture its deep structural meaning [43].

The interviews were recorded, transcribed verbatim and read several times to become familiar with the text. The qualitative software NVivo 10 was used for coding and analysing. Meaning units corresponding to the aim of the study were extracted, coded and grouped into sub-categories, categories and the theme. In order to strengthen credibility, the analysis was conducted independently by each of the authors, after which they discussed and achieved agreement about the findings [44].

## Results

The analysis revealed the theme Knowledge and communication lead to participation in the area of CAM based on three categories; CAM use, Communication and Self-care as well as fourteen sub-categories. The findings are presented under each category (Table 2).

**Table 2:** **Theme, categories and sub-categories**

Sub-categories	Categories	Theme
Changed dietary habits	CAM use	Knowledge and communication promote patient participation in the area of CAM
Factors that influence CAM use		
Experience of CAM		
Trust in CAM		
No need of CAM		
Doubtful about CAM		
Science and safety		
Perception of doctors’ and nurses’ attitude to CAM	Communication	
The relationship with the doctor		
Revealing CAM use		
Wishing for a dialogue about CAM		
Making one’s own choice	Self-care	
Wishing to live as normally as possible		
Searching for and obtaining CAM knowledge		

### CAM use

The category CAM use comprised the sub-categories: Changed dietary habits, Factors that influence CAM use, Experience of CAM, Trust in CAM, No need of CAM, Doubtful about CAM as well as Science and safety.

Some of the respondents considered that an important part of CAM was changed dietary habits, such as excluding gluten, lactose, dairy products, sugar, flour, E-numbers, raw meat and reducing calorie intake. They reported an improvement in their bowel disease as a result of various dietary changes but that they had not received sufficient support from HCPs in implementing the changes.

*I have been told that it doesn’t matter what you eat which sounds quite absurd to someone who suffers from bowel disease.*

The respondents mentioned several factors that influence CAM use such as side effects or when conventional treatment failed to produce the desired effect. They did not want to use corticosteroids and perceived taking many conventional medications as traumatic, although they were prepared to try everything, as long as it had a positive effect.

*Anything that will make me better is OK.*

The respondents perceived the HCPs as interested and open to CAM but lacking in knowledge and that they therefore needed information and education about CAM.

*The Health Authority ought to provide HCPs with information about CAM on a regular basis.*

CAM provided an opportunity for the body to heal itself and regain its balance. The respondents believed it important to have a holistic view of the human being and that CAM was perceived as exciting and popular, which was mainly due to reports in the media. Some considered CAM expensive and therefore did not use it to any great extent.

*But also the fact that CAM seems to be accepted in society is largely due to the media.*

In the respondents’ view there is a generational gap, younger people being more open to CAM, searching for information and having greater demands.

*I think that younger persons place different demands and perhaps search for information themselves on the Internet.*

*I think that older people have greater confidence in the doctor’s knowledge about medications and thus are slower* [to adopt CAM].

The respondents had experience of CAM. The majority had tried some type of dietary change during their illness in an effort to alleviate the disease, for example excluding carbohydrates, sugar and products containing solanine, such as potatoes, paprika and tomatoes. The respondents had boiled Chinese herbs, which they drank as tea and some had tried Probiotics with varying results.

*I don’t know if it was* [probiotics] *but I think so. It was really strong and I experienced a great difference at the beginning.*

Some of the respondents found massage and yoga effective for reducing stress and increasing their energy levels, thus alleviating IBD symptoms.

*It reduces the pain, the blood circulation is activated by the massage and I feel better and more relaxed, less stressed and my breathing is calmer.*

A number of respondents had used aloe vera juice and found it beneficial and a few had tried acupuncture but did not find that it had any effect on the IBD. None of the respondents who had tried CAM at least once for their IBD had been recommended to do so by HCPs but had searched for the information, initiated and paid for the treatment on their own initiative.

The respondents had trust in CAM, considered it valuable and that it had a place in healthcare. Some of them expressed that they experienced CAM as positive in that it reduced the negative effect of, for example, stress.

*I don’t think that CAM has any influence on the disease as such but on things around it.*

Some respondents had a special interest in CAM and had attended courses on the subject. They argued that certain CAM methods were more accepted than others and that it was definitely worth trying.

*You have to proceed by trial and error, but it’s absolutely worth trying. I sort of have no choice. I also think that it’s possible to prevent, support.*

Some of the respondents stated that they had no need of CAM and trusted their doctor. They did not miss CAM, nor did they search actively or ask HCPs for information about it.

*I’ve never considered trying CAM because I trust the health services, that they know what they are doing, it’s as simple as that.*

Some of the respondents mentioned having an ambivalent attitude, being doubtful about CAM, skeptical and not interested in using CAM methods. Others reported feeling doubtful about herbal treatment. Uncertainty as to whether CAM should be incorporated into healthcare also emerged.

*Then there are things that I haven’t tried, herbal medicines and such, I don’t trust them as much, because I have too little knowledge about them. My gut feeling is that there’s too much humbug in that area and a lot of desperation.*

With regard to science and safety, the respondents wanted scientific studies so that CAM would become evidence based. They were concerned that unscientific CAM methods and poorly qualified CAM practitioners could lead to *a* deterioration of the disease. They expressed an interest in testing different CAM methods provided they were based on scientific evidence and a few were positive towards participating in a clinical trial to test them.

*I think that it would be good if somebody carried out a study, so that I could have the opportunity to test some of it at a later stage. I can definitely envisage taking part.*

The respondents argued that it would feel safer to test CAM if it was offered by the health services. They respected the fact that HCPs have to work with evidence-based methods and stated that they would be positive towards working with CAM when research-based evidence becomes available.

*It’s easier to say Yes to something that you’re offered in the hospital than in some room located in a basement.*

The respondents were of the opinion that natural and health-promoting remedies were not perceived as harmful but that care should be exercised when taking them.

*Those health-promoting remedies, it’s mainly in order to test them. I don’t think they can harm you.*

### Communication

The category Communication was based on the following sub-categories: Patients’ perceptions of doctors’ and nurses’ attitudes to CAM, The relationship with the doctor, Revealing CAM use and Wishing for a dialogue about CAM.

The respondents perceived that the doctors and nurses lacked knowledge and were reluctant to talk about CAM and therefore considered the care to be inadequate. They reported having received no support from the healthcare service in terms of CAM use and that it was unusual for HCPs to discuss CAM. Some mentioned not having been taken seriously as well as treated in a disparaging manner when trying to discuss CAM. They were also critical of the dietary advice provided, arguing that HCPs should discuss diet more often with patients suffering from IBD.

*HCPs say: if you experience no adverse effect from it, you can go ahead, but we don’t exactly support you either.*

*HCPs don’t talk about CAM of their own accord.*

*When I became a vegetarian I was sneered at.*

The respondents considered that doctors were more sceptical than nurses when discussing CAM and that they received a better response from the latter. However, a few described HCPs’ attitude to CAM as dependent on the individual as opposed to the group and that there was no difference between nurses’ and doctors’ attitudes. The respondents claimed that doctors remained more faithful to their education and that HCPs did not see CAM as a threat. Nevertheless, a few had the impression that HCPs did not believe in CAM and that it was difficult to influence their negative attitude.

*I think there’s a difference in nurses’ and doctors’ attitudes to CAM. Doctors have more to defend and are more faithful to their education.*

There were also some respondents who regarded the relationship with the doctor as important. They trusted her/him and appreciated being allowed to participate in their care, as they wished to be actively involved in the discussion about their disease and treatment. However, they also stated that they complied with the doctor’s advice.

*I believe in participation, it’s incredibly important to me because I believe that everything has a better effect if you’re involved.*

*I’m very careful about ensuring that I know exactly what is happening to me and my body and therefore I’m actively involved in the discussions with my doctor.*

On the question of whether to reveal their CAM use, some did while others did not. Reasons for not revealing their CAM use was feeling ashamed, the knowledge that their doctor did not believe in CAM, respect for the doctor and the fact that CAM is not a part of health*c*are. They did not mention it unless the HCPs asked them; it was up to the patient to decide whether or not to talk about it.

*You feel ashamed, perhaps you have done something that is not quite OK or you don’t know what the doctor will think about it, maybe out of respect for the doctor.*

*I had a doctor who was a conventional doctor through and through. I knew from the start that it wasn’t even worth thinking about raising the subject.*

The respondents reported wishing for a dialogue about CAM and were open to discussing it as well as wanting to obtain support and help with CAM treatment. They found it easier to discuss with nurses than with doctors, as they considered the former to have a more relaxed attitude to CAM, which was why they sometimes only told nurses about their CAM use.

*I want to change my diet completely, but I haven’t had enough energy to tackle it. I would do it if I could get support and help* [from the HCPs]*.*

*But nurses are a bit more open to it* [CAM]*. It was easier to have a dialogue with them about it.*

### Self-care

The category Self-care comprised the sub-categories: Making own choices, Living as normally as possible and Searching for and obtaining CAM knowledge.

The respondents considered that making one’s own choices, being able to influence and doing something oneself were important, believing this to be the reason some turned to CAM.

*I think having an opportunity to influence one’s own situation makes people feel good. Take dietary change as an example, I think it makes a difference that you can sort of control it yourself.*

The respondents wished to live as normally as possible, did not want to change their lif*es*tyle but to be able to eat the same food as their family and friends. They were interested provided it was a CAM method they could easily adapt to, as they found it difficult to keep to a diet in everyday life and some did not have enough strength to undertake major dietary changes.

*I like to eat the same as my family and friends when we are out together.*

*It’s difficult to stick to such a diet on working days. I gradually went back to my previous diet and then it became worse again.*

The respondents searched for and obtained CAM knowledge via the Internet if and when they needed it. Some talked to and shared experiences with other patients.

*You try to talk to other patients to find out their experiences.*

## Discussion

The experiences of patients suffering from IBD demonstrate that knowledge and communication about CAM use in the context of healthcare promote patient participation in the area of CAM.

The main finding was that the respondents perceived that HCPs were reluctant to discuss CAM, as well as treating them in a disparaging manner and not taking them seriously when they raised the subject. Interestingly enough, this finding is in contrast to our earlier research, which indicated that HCPs believed that they emphasized the importance of the need to treat patients with respect when informed about their CAM use [45]. Studies have shown that HCPs lack education about CAM [39, 40, 45], which could be one reason patients’ interest in it was not taken seriously.

In Sweden, patients can report dissatisfaction with healthcare to the Patients*’* Advisory Committee (Patientnämnden). In the period 2006–2007, 13% of reports concerned patients being encountered in a wrong or disrespectful manner, which constituted the fourth most common complaint. How often such complaints were related to CAM use was not reported [46]. The result of the present study indicates a lack of communication between HCPs and patients with IBD. Some patients informed the HCPs about their CAM use while others did not. The reasons for not disclosing CAM use was shame*,* anticipation of disapproval or non-inquiry on the part of the doctor. This result is in line with previous research on CAM use in patients with cancer [47–49]. IBD professionals considered it crucial to create trust and a good relationship with the patient and for this reason did not ask about CAM, as they feared that such questions could have a negative impact on the relationship [45]. The results of the present study indicate the opposite, namely that patients suffering from IBD wanted to be asked about CAM use, but that if HCPs fail to do so, the patient her/himself will not mention it.

Patients with IBD do not communicate freely with their physicians about CAM [50]. In this study the respondents wanted a dialogue with HCPs about CAM. Some of the respondents found it easier to discuss CAM treatment with a nurse than with a doctor. The requirements of the Nurses-European Crohn’s and Colitis Organization (N-ECCO) include, among other things, the needs of patients’ with IBD, developing an empathetic and active listening role as well as providing essential IBD-related information and holistic support [51]. This clearly indicates that the role of IBD nurses is valuable in communication about and monitoring of CAM use. The patients need information about their disease. A full ten years after diagnosis, many patients with IBD were of the opinion that they had not received sufficient information about issues they now considered would have been essential to have discussed at the time of diagnosis [52].

Dietary changes are not clearly stated in NCCAM*’*s definition of CAM, although in this study the respondents expressed that dietary change was a part of CAM. Some patients feel dietary manipulation may be beneficial for them. These results are in line with Zallot et al*.,* who demonstrated that patients suffering from IBD try to implement dietary changes and avoid certain foods. Fifty-eight percent of their respondents believed that food played a role in disease relapse [53]. The respondents stated that HCPs were not supportive when it came to implementing dietary changes in daily life. One reason could be the lack of evidence of therapeutic benefit of dietary interventions in IBD, thus studies of such interventions are urgently required [54, 55]. However, research has revealed that a high protein diet, especially one rich in animal protein, may be associated with an increased risk of IBD [56–59]. In the present study the respondents considered self-care vital, wanted to live as normally as possible and placed great value on making their own decisions. A Canadian study showed that patients with IBD and Irritable Bowel Syndrom (IBS) used diet as a behavioural factor to control and minimize their symptoms [60]. Zallot et al. found (2013) that a large number of patients with IBD avoided certain foods and reported that food had a major impact on their social life [53].

The respondents in the present study wished for dialogue about CAM to enable their participation in the treatment. Healthcare in Sweden is regulated by the Health Care Act [6] *,* which states that HCPs should provide the patient with information and involve her/him in decisions about care and treatment*,* defined as patient participation. However, for the patient, participation means “being involved in a life situation” rather than just taking part in decisions about care and treatment [61]. This is something HCPs need to consider in order to meet patient expectations by improving patient participation in the area of CAM and creating opportunities for CAM discussions.

### Methodological considerations

The qualitative study design allowed the respondents to talk freely about their experiences of CAM. The analyses reveal their experiences by means of their words and statements. To strengthen the internal validity, criteria for trustworthiness were applied; credibility, transferability, dependability and conformability [44].

Credibility was strengthened by selecting respondents of different ages, gender, disease duration and with a diagnosis of UC or CD. The respondents were informed that participation was voluntary and that they could withdraw at any time. Furthermore, three of the authors (AL, BF, LO) independently read the interviews, coded them and discussed the analysis until consensus was achieved. Transferability was ensured by the rich descriptions of the phenomenon, even in the shortest interview*,* which lasted for 15 minutes. The context was described, which also supports transferability. Dependability was enhanced by a description of the research process and design. The last area of trustworthiness mentioned by Lincoln and Guba [44] is conformability; i.e. the researcher should remain objective. The first author (AL) who conducted the interviews has been employed as an IBD nurse and worked with such patients for 15 years but was not involved in the care of the respondents. To prevent bias in the analysis due to pre-understanding, all authors independently read and analysed the text, after which disagreements were discussed and consensus achieved. However, in qualitative studies, the credibility of the researcher is of major importance, thus the present author’s pre-understanding can be considered a strength as it ensured knowledge and comprehension of the context [43].

## Conclusion

The experiences of patients with IBD demonstrated that HCPs were reluctant to discuss CAM, and some treated them in a disparaging manner and did not take them seriously when they raised the subject. They wanted to be asked about CAM in order to engage in a dialogue. HCPs must be aware of this, so that they can meet and understand patient needs. The finding that patients considered it easier to discuss CAM with nurses than physicians highlights the important role of IBD nurses in providing information about and monitoring CAM use. HCPs also need to understand that dietary changes are an important aspect of CAM treatment for many patients who suffer from IBD. Further research in these areas is needed.
